# Long Alkylene Spacers Promote Structural Ordering and Proton Transport in Phosphonic Acid–Based Polymer Electrolyte Membranes

**DOI:** 10.1021/acsomega.5c10883

**Published:** 2026-01-16

**Authors:** Itsuki Takashima, Takato Kajita, Takenori Nakayama, Mio Nishimoto, Haruka Tanaka, Atsushi Noro

**Affiliations:** † Department of Molecular & Macromolecular Chemistry, Graduate School of Engineering, 12965 Nagoya University, Furo-Cho, Chikusa-Ku, Nagoya 464-8603, Japan; ‡ Institute of Materials Innovation, Institutes of Innovation for Future Society, Nagoya University, Furo-Cho, Chikusa-Ku, Nagoya 464-8601, Japan; § Research Center for Net-Zero Carbon Society, Institutes of Innovation for Future Society, Nagoya University, Furo-Cho, Chikusa-Ku, Nagoya 464-8601, Japan

## Abstract

Next-generation polymer electrolyte fuel cells (PEFCs) require polymer electrolyte membranes (PEMs) capable of operating at temperatures above the boiling point of water (100 °C) and under low-humidity below 40% RH. In this study, we synthesized poly­(8-(*p*-styryl)-1-octanephosphonic acid) (soPA), a polymer bearing phosphonic acid groups on the side chains connected via eight-carbon alkylene spacers. soPA was insoluble in water and formed a highly oriented lamellar phase-separated nanostructure with a 2.9 nm domain spacing, consisting of a hydrophobic phase formed by the alkylene spacers and the polystyrene backbone, and a hydrophilic phase containing the phosphonic acid groups. Despite its lower acid group density compared with poly­(4-(*p*-styryl)-1-butanephosphonic acid) (sbPA) with shorter four-carbon spacers, the soPA membrane exhibited higher conductivities than sbPA. For instance, soPA achieved conductivities of 4.4 and 7.5 mS cm^–1^ at 120 °C under 20% and 40% RH, respectively, values which are approximately 4 and 2.9 times higher than those of sbPA. These enhanced conductivities of soPA can be attributed to the higher morphological and molecular ordering induced by the nanophase separation as well as the greater freedom of motion of the phosphonic acid groups provided by the flexibility of the longer alkylene spacers, which facilitate more efficient proton transfer between them.

## Graphical abstract

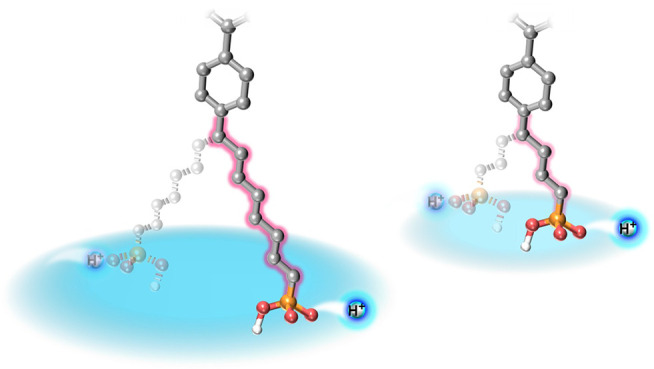

## 1 Introduction

Fuel cells that use hydrogen gas as fuel are clean and environmentally friendly power generation systems as they produce only water as a byproduct. Therefore, fuel cells have attracted increasing attention in recent years, driven by the global trend toward a net-zero carbon society.^1–3^ In particular, polymer electrolyte fuel cells (PEFCs), which employ polymer electrolyte membranes (PEMs) as electrolytes, have already been implemented in commercially available fuel cell vehicles (FCVs)^4–6^ and household fuel cell cogeneration systems.^4,7^

Perfluorosulfonic acid (PFSA) membranes, such as Nafion,^8,9^ are representative PEMs used in PEFCs. PFSA membranes exhibit high conductivities (≥100 mS cm^–1^) under low temperatures (<100 °C) and high humidities (60–90% RH),^10,11^ primarily due to the Grötthuss mechanism, in which protons hop through water molecules.^12,13^ However, since PFSA membranes are composed of per- and polyfluoroalkyl substances (PFAS), they are highly resistant to environmental degradation and thus impose a significant environmental burden.^14,15^

Meanwhile, hydrocarbon-based PEMs without fluorine atoms have also been actively developed.^16–26^ For example, Pemion membranes are known as a semicommercial hydrocarbon PEM.^27,28^ However, sulfonic acid polymer membranes generally exhibit low conductivity and easily undergo degradation under the operating conditions required for next-generation PEFCs, namely, temperatures above 100 °C (the boiling point of water) and humidity below 40% RH.^29–31^ On the other hand, the reaction rates in the fuel cells are relatively slow at temperatures below 100 °C, requiring higher platinum loadings in the catalyst layer of PEFCs. To reduce the required platinum loading, the development of PEMs that can operate under the conditions for next-generation PEFCs is desired.

Phosphonic acid–based polymers, which are more chemically stable at high temperatures and low humidity than sulfonic acid–based polymers because the C–P bond in the phosphonic acid group exhibits higher chemical stability than the C–S bond in the sulfonic acid group,^32,33^ are promising alternatives.^34–48^ Representative examples include poly­(vinylphosphonic acid)^49,50^ and poly­(*p*-styrenephosphonic acid) (sPA).^43,50^ Recently, we synthesized poly­(4-(*p*-styryl)-1-butanephosphonic acid) (sbPA), a polystyrene-based polymer bearing phosphonic acid groups on side chains via alkylene spacers with neither ether nor ester bonds.^51^ While an sPA membrane, which lacks spacer groups, easily dissolves in water, the sbPA membrane remained insoluble in water, despite the absence of cross-linking. Furthermore, under high temperature and low-humidity conditions (e.g., 120 °C and 20% RH), the sPA membrane exhibited a low conductivity of 0.027 mS cm^–1^, whereas the sbPA membrane exhibited a much higher conductivity of 1.1 mS cm^–1^, approximately 40 times greater than that of sPA. The insolubility and the improved conductivity of sbPA were attributed to the presence of hydrophobic alkylene spacers between the polymer backbone and the phosphonic acid groups. The alkylene spacers facilitate proton transfer between adjacent acid groups by allowing them to approach each other more closely in a manner similar to a vehicle-type mechanism.^52,53^

However, the conductivity of the sbPA membrane was not sufficiently high for practical PEFC applications. Our previous studies suggested that higher conductivity might be achieved by increasing the length of the alkylene spacers. The conductivities of polymers with different spacer lengths have been evaluated in some previous studies. For example, poly­(2-(4-vinylbenzyloxy)­ethylphosphonic acid) and poly­(6-(4-vinylbenzyloxy)­hexylphosphonic acid) exhibited similar conductivities (∼0.3 mS cm^–1^) at a high temperature of 140 °C under a dry nitrogen atmosphere (nominally anhydrous condition), regardless of spacer length.^54^ A comparison of poly­(ω-(4-vinylphenoxy)­alkylphosphonic acid) with spacer lengths containing four, six, or eight carbon atoms revealed the polymer with a four-carbon spacer exhibited slightly higher conductivities at 80 °C and 30–95%RH than those with six- or eight-carbon spacers.^55^ This behavior has been attributed to the higher ion-exchange capacity (IEC) of polymers with shorter spacers.

In contrast, for poly­((acryloyloxy)­alkylphosphonic acid) with spacer length containing two, four, or six carbon atoms, where the polymers were cross-linked to prevent dissolution in water, the polymer with a six-carbon spacer exhibited higher conductivities at 80 °C and 30–80% RH than did those with two- or four-carbon spacers, despite having the lowest acid group density.^56^ All these results indicate that the relationship between spacer length and conductivity has not been fully elucidated. In particular, the effect of spacer length on proton conduction in phosphonic acid–based polymers in uncross-linked PEMs that are insoluble in water has not been well investigated under conditions of high-temperature (above 100 °C) and low-humidity.

Based on the above discussion, we synthesize poly­(ω-(*p*-styryl)-α-alkanephosphonic acid) bearing phosphonic acid groups through longer alkylene spacers on the side chains than those of sbPA, which have a four-carbon spacer. Specifically, diethyl 8-(*p*-styryl)-1-octanephosphonate monomer with an eight-carbon alkylene spacer is synthesized and subsequently polymerized to yield poly­(diethyl 8-(*p*-styryl)-1-octanephosphonate) (soPAdE). Thereafter, the phosphonate ester groups in soPAdE are deprotected to afford poly­(8-(*p*-styryl)-1-octanephosphonic acid) (soPA; Figure 1a). We evaluate the conductivities of soPA membranes under operating conditions required for next-generation PEFCs (120 °C and below 40% RH) and compared them with those of sbPA membranes (Figure 1b) to provide fundamental insights into the effect of alkylene spacer length on the phase-separated structure and membrane properties.

**Figure 1:**
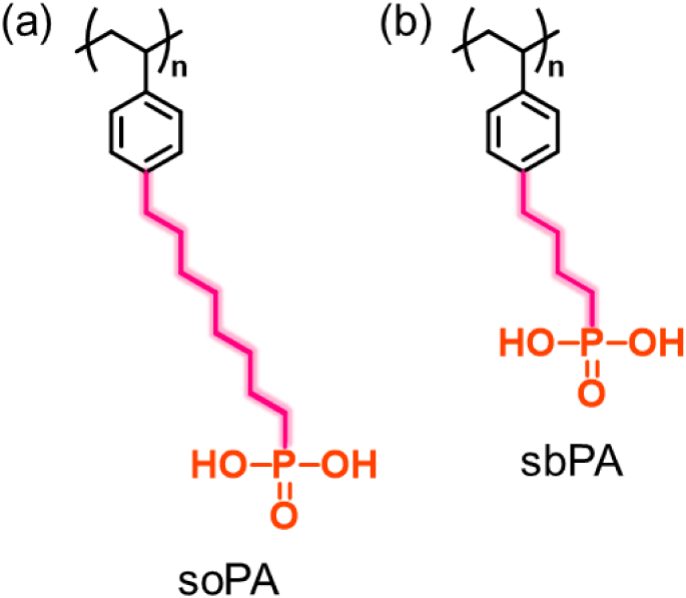
Chemical structures of (a) poly­(8-(*p*-styryl)-1-octanephosphonic acid) (soPA) and (b) poly­(4-(*p*-styryl)-1-butanephosphonic acid) (sbPA). Pink lines indicate the alkylene spacer groups.

## 2 Experimental Section

### 2.1 Materials

*p-*Bromostyrene was purchased from TCI, and the stabilizer was removed by passing it through an alumina column which was purchased from Aldrich (grade: activated, basic, Brockmann I) prior to use. 1,8-Dibromooctane (TCI) was dried with 3A molecular sieves (Nacalai Tesque, Inc.) prior to use. Triethyl phosphite (Aldrich), dehydrated tetrahydrofuran (THF, Fujifilm Wako Pure Chemical Corporation), methanol (Fujifilm Wako Pure Chemical Corporation), chloroform (Kanto Chemical Co., Inc.), *n*-hexane (Kanto Chemical Co., Inc.), and ethyl acetate (Kishida Chemical Co., Ltd.) were used as received. *n*-Butyllithium in *n*-hexane (1.6 mol L^–1^, Kanto Chemical Co., Inc.), 2,2-azobis­(isobutyronitrile) (AIBN, Kishida Chemical Co., Ltd.), 2-(dodecylthiocarbonothioylthio)-2-methylpropionic acid (DDMAT, Aldrich), which is a reversible addition–fragmentation chain transfer (RAFT) agent, and bromotrimethylsilane (Aldrich) were also used as received. An sbPA membrane used as a control sample was prepared in the same manner as previously reported.^51^ The number-average molecular weight (*M*_n_) and dispersity (*Đ*) of sbPA before deprotection were 130k and 1.82, respectively. Both *M*_n_ and *Đ* were calibrated using polystyrene standards.

### 2.2 Synthesis of Diethyl 8-(*p*-Styryl)-1-Octanephosphonate

Following the previously reported synthetic scheme for diethyl 4-(*p*-styryl)-1-butanephosphonate,^51^ diethyl 8-(*p*-styryl)-1-octanephosphonate was synthesized by substituting 1,8-dibromooctane for 1,4-dibromobutane (Scheme 1a). *p*-Bromostyrene (1.3 g, 7.1 mmol) was dissolved in 30 mL of dried THF and cooled to −78 °C. A halogen-lithium exchange reaction was carried out by adding *n*-butyllithium in hexane (1.6 mol L^–1^, 4.9 mL, 7.8 mmol) to the mixture. Then, 1,8-dibromooctane (19 g, 71 mmol) was added to initiate a nucleophilic substitution reaction, and the reaction was quenched with methanol. Finally, yellowish oily *p*-(8-bromooctyl)­styrene (1.7 g, 80%) was obtained by removing the solvent and unreacted 1,8-dibromooctane through rotary evaporation followed by vacuum distillation.

**Scheme 1:**
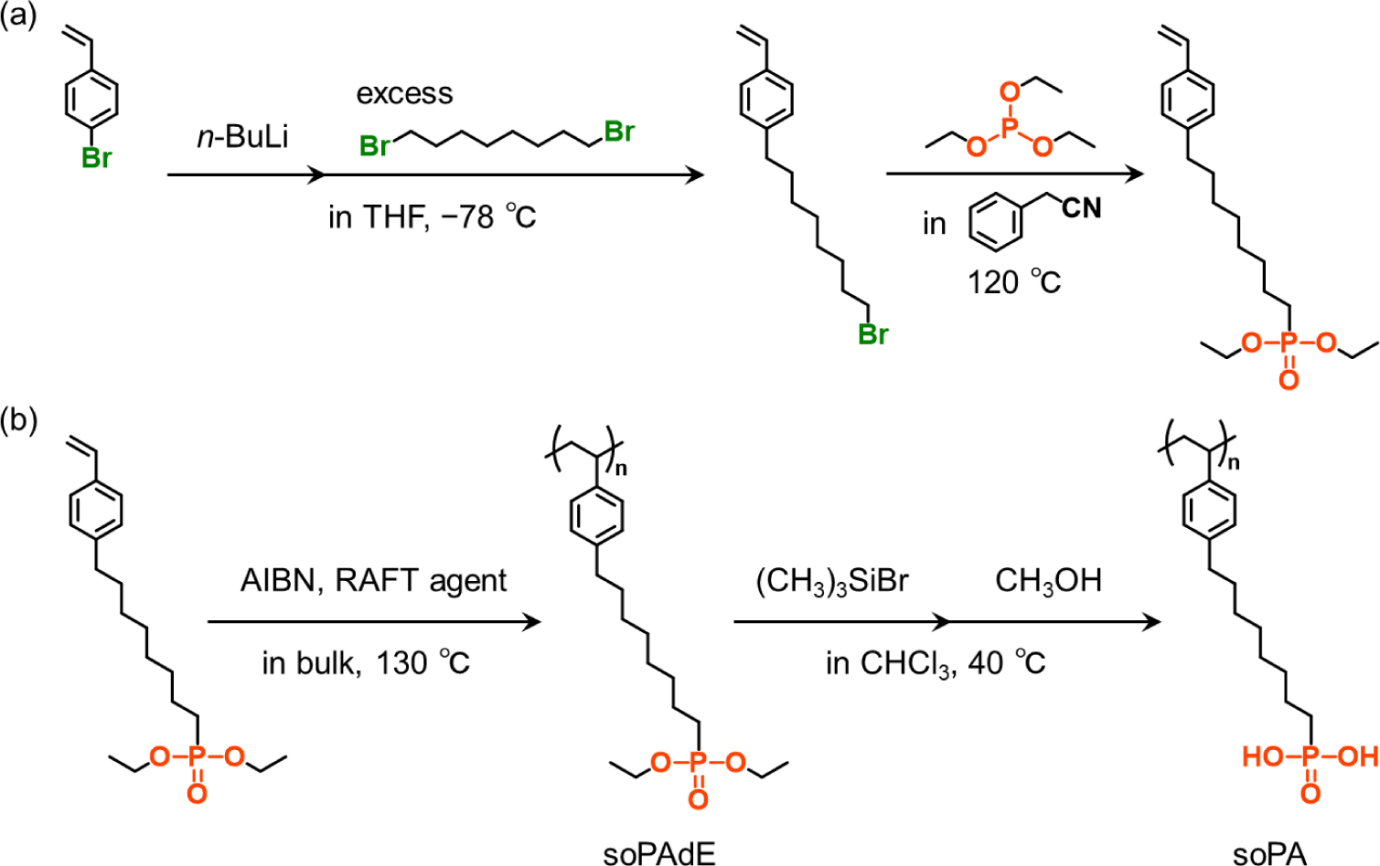
Synthesis of (a) Diethyl 8-(*p*-Styryl)-1-Octanephosphonate and (b) soPA.^a^

a Note that the RAFT agent residue group is omitted from the chemical structures for clarity.

To perform a Michaelis-Arbuzov reaction,^57^ *p*-(8-bromooctyl)­styrene (1.7 g, 5.7 mmol) and triethyl phosphite (1.9 g, 11 mmol) were dissolved in 1.3 g of phenylacetonitrile, and the solution was stirred overnight at 120 °C under a nitrogen atmosphere. The resulting solution was distilled, followed by performing silica gel column chromatography using chloroform, *n*-hexane, and methanol as eluents to afford yellowish oily diethyl 8-(*p*-styryl)-1-octanephosphonate (yield: 1.2 g, 60%).

### 2.3 Synthesis of Poly­(8-(*p*-Styryl)-1-Octanephosphonic Acid)

soPAdE was prepared by RAFT polymerization^58,59^ of diethyl 8-(*p*-styryl)-1-octanephosphonate, and soPA was prepared by treating soPAdE with bromotrimethylsilane, followed by transforming the phosphonate ester groups into the phosphonic acid group using methanol^60,61^ (Scheme 1b). Purified diethyl 8-(*p*-styryl)-1-octanephosphonate (1.0 g, 2.8 mmol), DDMAT (1.4 mg, 3.8 μmol), and AIBN (0.62 mg, 3.8 μmol) were mixed and purged with argon gas. The mixture was stirred at 130 °C for 2 h, and the polymerization reaction was terminated by cooling with liquid nitrogen to afford soPAdE (0.53 g, 53%).

The obtained soPAdE (0.53 g, containing 1.5 mmol of phosphonate ester groups) was dissolved in 4.5 mL of anhydrous chloroform, followed by adding bromotrimethylsilane (2.3 g, 15 mmol) and stirring the mixture at 40 °C overnight. The reaction mixture was treated with excess methanol, and the resulting solution was dialyzed against deionized water using a cellulose tube to purify the polymer. The dialyzed solution was dried to afford soPA as a white solid (0.42 g, 95%).

### 2.4 Characterization

To characterize the synthesized monomers, soPAdE, and soPA, ^1^H and ^31^P nuclear magnetic resonance (NMR) spectra were recorded using an AVANCE III HD 500 MHz spectrometer (Bruker). CDCl_3_ or THF-*d*_8_/D_2_O (1:1 by weight) were used as the deuterated solvents. Tetramethylsilane (TMS) was used as the internal standard for ^1^H NMR, and an 85% aqueous phosphoric acid (H_3_PO_4_) solution was adopted as the external standard for the ^31^P NMR.

Gel permeation chromatography (GPC) was performed to determine the number-average molecular weights (*M*_n_) and dispersity (*Đ*) of soPAdE. Both *M*_n_ and *Đ* were calibrated using polystyrene standards. An HPLC system equipped with an LC-20 AD HPLC pump (Shimadzu), a CTO-20A column oven (Shimadzu), an SPD-20A UV detector (Shimadzu), and two TSKgel GMH_HR_-M columns (Tosoh) was used for the GPC measurements. A THF/MeOH/H_2_O (weight fraction: 80/19/1) mixture containing a minor quantity of tetrabutylammonium bromide salts was used as the eluent.

Differential scanning calorimetry (DSC) was carried out using a Q2000 calorimeter (TA Instruments) at a heating rate of 10 °C min^–1^ under a nitrogen atmosphere over the temperature range of 50–230 °C to determine the glass transition temperature (*T*_g_) of soPA.

### 2.5 Preparation of Membranes

To prepare the soPA membrane, soPA (30 mg) was dissolved in 3 mL of a mixture of 2-propanol/basic water (pH ∼ 13) = 5/5 (w/w). The solution was cast at 65 °C for 1 day, and the produced membrane was then placed in acidic aqueous media (pH ∼ 1) at 60 °C for 1 h. Finally, the dried membrane was hot-pressed at 130 °C for 1 min.

### 2.6 Measurements

The water insolubility of the PEM was evaluated by immersing the PEM in pure water at 60 °C for 3 h and comparing the weight of dried PEM before and after water immersion.

Water uptake (WU) and thickness swelling ratio (SR_*t*_) were also determined. First, the weight (*W*_dry_) and thickness (*t*_dry_) of the sufficiently dried PEM were measured, and the PEM was then soaked in pure water at 60 °C for 3 h. The weight (*W*_wet_) and thickness (*t*_wet_) of the membrane were measured after wiping away the surface water from the PEM, and WU and SR_*t*_ were calculated using eqs 1 and 2, respectively.

1 $$\mathrm{WU}=\frac{W_{\mathrm{wet}}-W_{\mathrm{dry}}}{W_{\mathrm{dry}}}$$

2 $${\mathrm{SR}}_{t}=\frac{t_{\mathrm{wet}}-t_{\mathrm{dry}}}{t_{\mathrm{dry}}}$$

Transmission electron microscopy (TEM) was performed to directly observe the nanostructure of the soPA membrane. Ultrathin sections for the observation were prepared by microtomy, followed by staining with osmium tetroxide (OsO_4_). TEM observations were performed using an HT-7700 electron microscope (Hitachi) operated at an accelerating voltage of 100 kV.

To investigate the nanostructure of the soPA membranes in reciprocal space, X-ray scattering measurements were also performed at ambient temperature and humidity using an R-AXIS IV X-ray diffractometer (Rigaku). The X-ray wavelength (λ) and camera length were set to 0.154 nm and 200 mm, respectively.

The conductivities of the soPA membrane were evaluated by alternating current impedance spectroscopy using a VSP-300 potentiostat/galvanostat (BioLogic Science Instruments) with an imposed 50 mV oscillatory signal throughout a frequency interval of 1 Hz and 7 × 10^6^ Hz, as previously reported.^26,51,62,63^ A cell using platinum-electrodes was placed in an SH-242 benchtop environmental chamber (ESPEC Corp.). The measurements were conducted at 80 °C and 10–100% RH or at 120 °C and 10–40% RH. The temperature and humidity were monitored using a Testo 440 thermohygrometer equipped with a robust humidity/temperature probe (Testo SE & Co. KGaA). The conductivity (σdc) was determined from the Nyquist plots, as previously reported (see also the Supporting Information for details of the measurements).

## 3 Results and Discussion

### 3.1 Synthesis of Diethyl 8-(*p*-Styryl)-1-Octanephosphonate

Diethyl 8-(*p*-styryl)-1-octanephosphonate monomer was obtanied by a two-step reaction summerized in Scheme 1a. Figure 2 shows ^1^H NMR spectra of the products obtained after the first and second steps. In the spectrum of the product isolated after the first reaction step, signals attributable to the vinyl protons (a′–c′) appeared at 5.2, 5.7, and 6.7 ppm, and signals corresponding to the aromatic protons (d′ and e′) were observed at approximately 7.1 and 7.3 ppm. The methylene protons adjacent to the phenylene group (f′) and the bromo group (m′) also gave signals at 2.6 and 3.4 ppm, respectively, whereas signals originating from the other methylene protons (g′–l′) appeared at 1.3–1.8 ppm. The integral ratios of the signals attributable to all methylene protons were consistent with the theoretical values, confirming the successful synthesis of *p*-(8-bromooctyl)­styrene.

**Figure 2:**
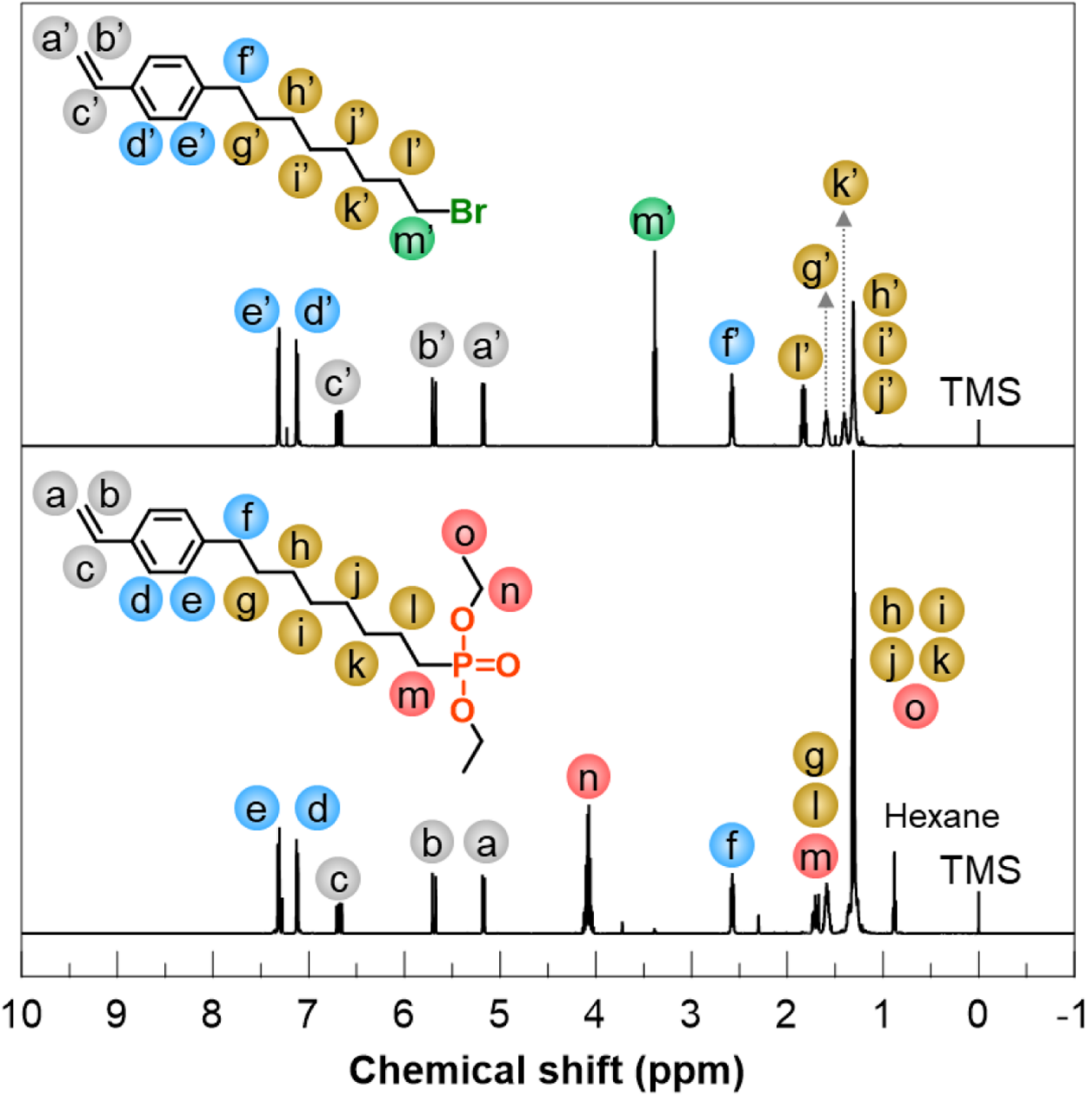
^1^H NMR spectra of *p-*(8-bromooctyl)­styrene (top) and diethyl 8-(*p*-styryl)-1-octanephosphonate (bottom) in CDCl_3_.

In the ^1^H NMR spectrum of the compound obtained after the second reaction step, the chemical shifts and integral ratios of signals corresponding to protons on the styryl group (a′–e′ and a–e) and the benzylic position (f′ and f) were nearly identical. Meanwhile, the signal at 3.4 ppm corresponding to the methylene protons (m′) adjacent to the bromo group disappeared. Instead, a new signal appeared at approximately 1.7 ppm, attributed to the methylene protons (m) adjacent to the phosphorus atom. Additionally, signals newly observed at approximately 4.1 and 1.3 ppm were assigned to protons on the oxygen-adjacent methylene group on the phosphonate ester group (n) and the methyl protons (o) on the phosphonate ester group, respectively. The integral ratio of the signal n was also twice that of the benzylic methylene protons (f). Furthermore, the ^31^P NMR spectrum of the purified final product exhibited a signal at 33 ppm, which is characteristic of the phosphorus atom in the phosphonate ester (Figure S1). These results indicate that diethyl 8-(*p*-styryl)-1-octanephosphonate was yielded as the final product.

### 3.2 Synthesis of Poly­(8-(*p*-Styryl)-1-Octanephosphonic Acid) (soPA)

soPAdE was synthesized via RAFT polymerization of diethyl 8-(*p*-styryl)-1-octanephosphonate, followed by deprotection of the phosphonate ester to obtain soPA (Scheme 1b). Figure 3 shows the ^1^H NMR spectra of the samples obtained after polymerization and after deprotection, and Figure 4 shows ^31^P NMR spectra of these samples. The signals at 5.2, 5.7, and 6.7 ppm, which were derived from the vinyl protons (Figure 2, bottom), disappeared in the spectrum of the sample after polymerization. In addition, the GPC chromatogram of the polymerization product was unimodal (Figure S2), indicating that soPAdE was obtained. The *M*_n_ and *Đ* of soPAdE, calibrated using polystyrene standards, were 55 k and 1.96, respectively. The ^31^P NMR spectrum of soPAdE exhibited a signal at 33 ppm, which was almost the same chemical shift as that in the ^31^P NMR spectrum of diethyl 8-(*p*-styryl)-1-octanephosphonate.

**Figure 3:**
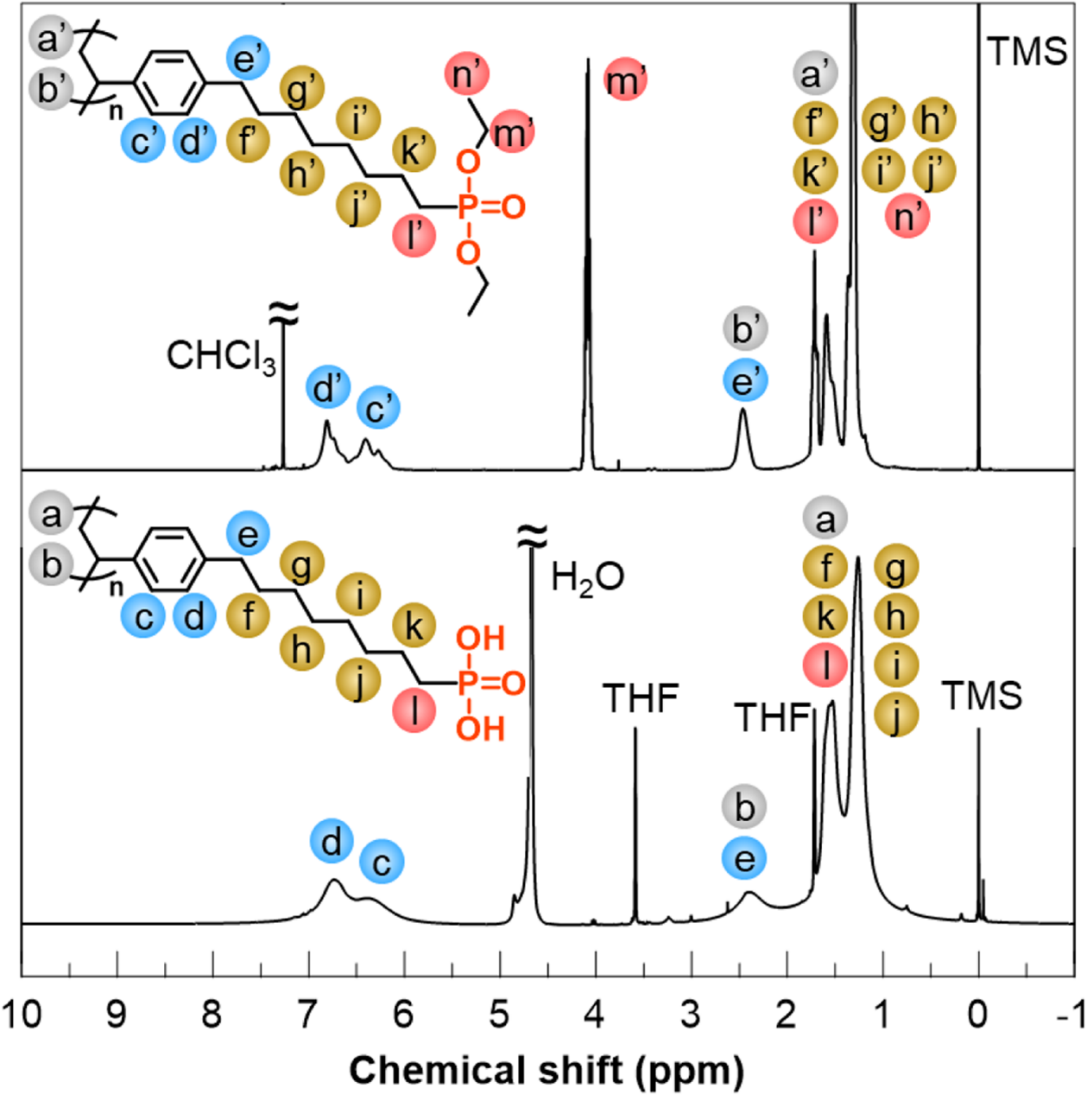
^1^H NMR spectra of soPAdE in CDCl_3_ (top), and soPA in THF-*d*_8_/D_2_O (bottom).

**Figure 4:**
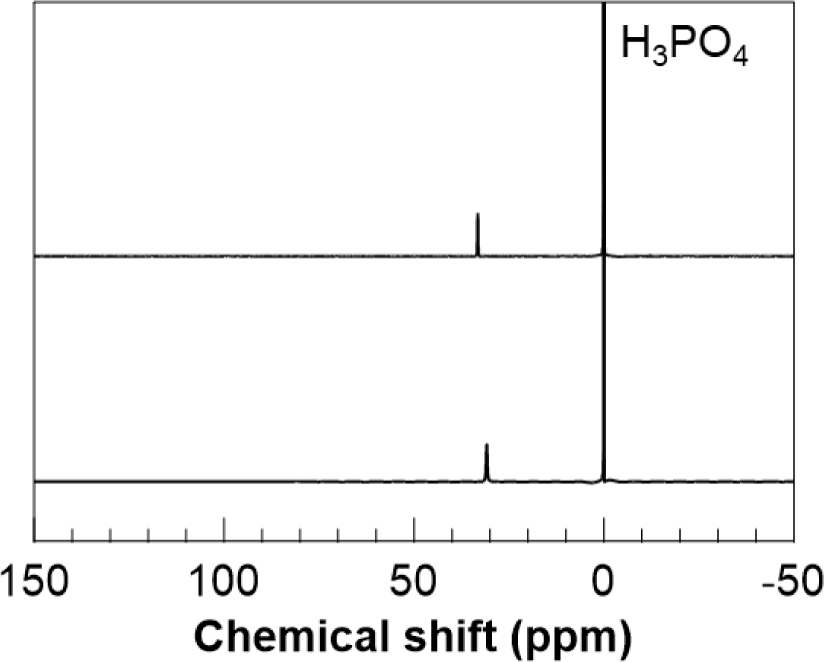
^31^P NMR spectra of soPAdE in CDCl_3_ (top), soPA in THF-*d*_8_/D_2_O (bottom).

The signals at approximately 1.3 and 4.1 ppm in the ^1^H NMR spectrum of soPAdE, which were attributed to the protons of the ethyl groups on the phosphonate ester groups (m′ and n′), almost completely disappeared in the ^1^H NMR spectrum of the product after deprotection. Furthermore, compared with the signal in the ^31^P NMR spectrum of soPAdE, the signal originating from the phosphorus atoms was shifted to 31 ppm on the upfield side in the ^31^P NMR spectrum of the product after deprotection. These spectral changes indicate that the deprotection of phosphonate ester groups in soPAdE proceeded nearly quantitatively. *T*_g_ of soPA was determined to be 183 °C, and the thermal degradation behavior was similar to that of sbPA. (see also the DSC and thermogravimetric analysis (TGA) thermogram in Figures S3 and S4, respectively).

### 3.3 Insolubility of the soPA Membrane in Water

Water insolubility of the soPA membrane was evaluated by measuring the residual weight fraction, WU, and SR_*t*_ after immersion of the soPA membrane in pure water at 60 °C for 3 h. As shown in Figure S5, the soPA membrane was not soluble in water, a similar outcome as for the previously reported sbPA membrane. The residual weight fraction of the soPA membrane was higher than 99%, confirming that soPA is insoluble in water under conditions comparable those encountered during fuel cell operation, including emergency shutdown. Table 1 summarizes WU and SR_*t*_ values of the soPA and sbPA membranes. The WU of soPA was 32%, which was comparable to that of sbPA (41%). On the other hand, the soPA membrane exhibited a greater than 3-fold larger SR_*t*_ of 35% than that of the sbPA membrane (9.9%). It should be noted that the residual weight fraction of the soPA membrane remained higher than 99% after a Fenton test using 2 ppm of FeSO_2_ and 3.5% of H_2_O_2_ at 80 °C for 1 h, indicating the good oxidative stability of the soPA membrane.

**Table 1:** Characteristics of soPA and sbPA Membranes

Sample	*d*_A_ ^a^ (mmol g^^–1^^)	WU^b^ (%)	SR_*t*_ ^c^ (%)
soPA	3.4^d^	32	35
sbPA	4.2^d^,^51^	41 ^51^	9.9 ^51^

a Acid group density, which refers to the number of acidic groups per unit weight. The acid group densities of soPA and sbPA correspond to half of the theoretical ion exchange capacity (IEC).

b Water uptake caluculated by eq 1 The PEMs were soaked in water at 60 °C for 3 h and then thoroughly wiped to remove water on the surface of the samples.

c Thickness swelling ratio caluculated by eq 2 The PEMs were soaked in water at 60 °C for 3 h.

d Calculated from the monomer molecular weight and the progress of deprotection reaction estimated by ^1^H NMR measurements.

### 3.4 Nanostructure of the soPA Membrane

In a typical TEM micrograph of the soPA membrane (Figure 5a), the phase containing the alkylene spacer groups and the polystyrene backbone display a comparatively bright contrast, while the other phase containing the phosphonic acid groups are visualized with darker contrast due to OsO_4_ staining. The TEM image of the soPA membrane revealed a well-oriented lamellar phase-separated structure with a domain spacing (*D*) of approximately 2.8–3.0 nm. On the other hand, as previously reported, the TEM image of the sbPA membrane showed a poorly oriented lamellar or cylindrical phase-separated structure with a *D* of approximately 2.0–2.5 nm.^51^

**Figure 5:**
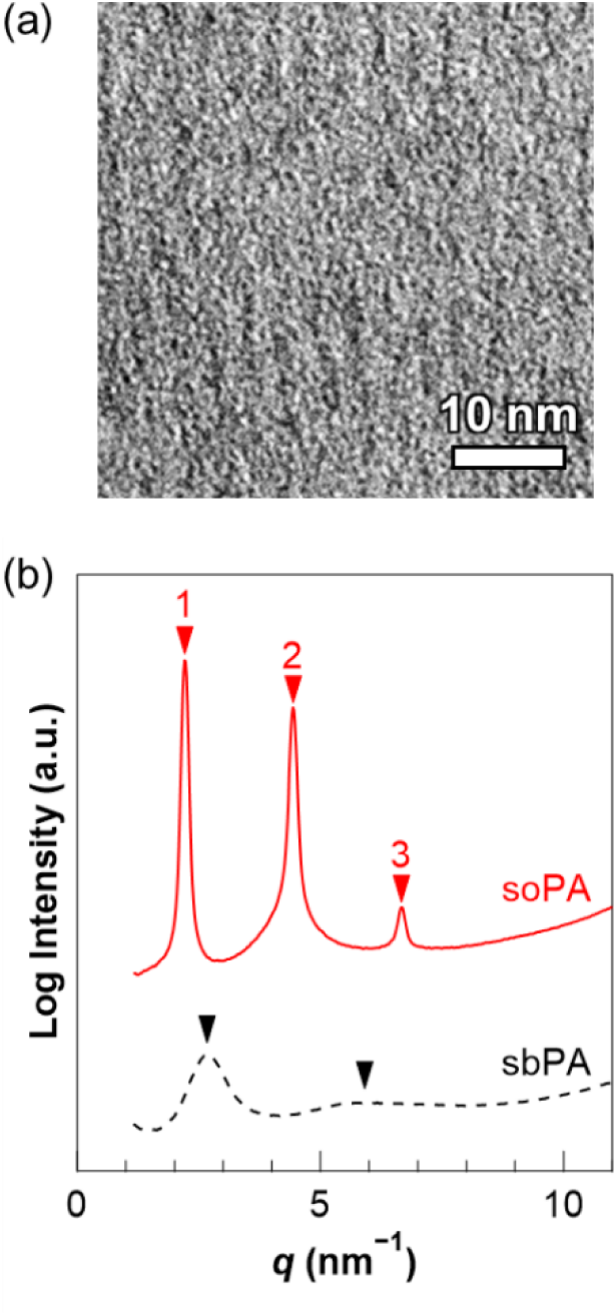
(a) TEM images of soPA membrane, where scale bar represents 10 nm. (b) X-ray scattering profiles of soPA (red solid line) and of sbPA (black dashed line) at ambient temperature and humidity.

In addition to TEM observations, we also performed X-ray scattering measurements of the soPA and sbPA membranes at ambient temperature and humidity (Figure 5b). In the profile of the soPA membrane, a primary peak (*q*_1_) was observed at a scattering vector (*q* = 4πsinθ/λ) of 2.2 nm^–1^. Secondary and tertiary peaks were also observed at relative positions approximately 2 and 3 times that of the primary peak (*i.e*., *q*_2_ ≈ 4.4 nm^– 1^ and *q*_3_ ≈ 6.6 nm^– 1^), respectively. Therefore, a highly oriented lamellar phase-separated structure was formed in the soPA membrane, and its *D* (= 2π/*q*_1_)^64^ was estimated to be 2.9 nm from the *q*_1_ value, which roughly agreed with the TEM image. On the other hand, the sbPA membrane exhibited a *q*_1_ at 2.7 nm^–1^, with only a secondary peak observed at approximately twice the position of *q*_1_. Both peaks were relatively broad, suggesting the formation of a poorly oriented lamellar or cylindrical phase-separated structure in the sbPA membrane. Assuming a lamellar structure, the *D* was estimated to be 2.4 nm, and under the assumption of a cylindrical structure, *D* (= (4/3)^1/2^ × 2π/*q*_1_)^65^ was estimated to be 2.7 nm.

Polymers bearing both ionic groups and hydrophobic alkyl chains within the monomer unit often exhibit phase separation between these two components even in the absence of crystallinity.^66^ Similarly, both soPA and sbPA did not exhibit the crystallinity, as evidenced by the absence of endothermic melting peaks in the DSC thermograms (Figure S3), and showed a phase-separated structure consisting of a hydrophobic phase containing the alkylene spacers and the polystyrene backbone, and a hydrophilic phase containing the phosphonic acid groups. It has been reported that the phase boundaries in the phase diagram of diblock copolymers with one charged block shift significantly toward the side of the smaller fraction of the charged block.^67^ By analogy, the phase diagram of the phosphonic acid polymers should resemble that of charged diblock copolymers, because the phosphonic acid groups behave as ionic species through facile dissociation protons and phosphonate anions. This analogy accounts for the lamellar structures observed in the soPA and sbPA membranes and the more highly oriented nanostructure of soPA, which arises from the larger effective *χN* associated with the longer alkylene spacer within the monomer unit compared with sbPA (see also the schematic illustration of the phase diagram in Figure S6), where χ and *N* are, respectively, the Flory–Huggins interaction parameter between the blocks and the overall degree of polymerization in the block copolymers. Furthermore, the larger *D* value of soPA directly reflects the longer alkylene spacer length compared with sbPA.

The relatively well-oriented lamellar structure of the soPA membrane allowed the hydrophilic domains to swell upon water uptake. In contrast, the hydrophilic domains in the sbPA membrane were easily surrounded by hydrophobic domains due to the poorly oriented morphology, which restricted swelling in water. Therefore, this structural difference accounts for the larger SR_*t*_ of soPA compared with sbPA.^68,69^

### 3.5 Conductivities of the soPA Membrane

To investigate the effect of the alkylene spacer length on conductivity, we measured the conductivities of the soPA membrane at 80 °C, which is the typical operating temperature of PEFCs, and at 120 °C, which corresponds to the operating temperature required for next-generation PEFCs and exceeds the boiling point of water. These results were also compared with the previously reported conductivities of the sbPA membrane. Figure 6a,b show the humidity dependence of σdcs at 80 and 120 °C, respectively, and the σdc values are summarized in Table S1 (see also the Nyquist plots for all measurements in Figure S7). The σdc of sbPA was 17 mS cm^–1^ at 80 °C and 100% RH, and the conductivity gradually reduced with decreasing humidity, reaching 0.056 mS cm^–1^ at 80 °C and 10% RH.^51^ On the other hand, despite the lower acid group density of soPA (Table 1) due to its longer alkylene spacer, the soPA membrane exhibited an approximately 2.4 times higher σdc of 40 mS cm^–1^ at 80 °C and 100% RH than that of the sbPA membrane. Furthermore, the influence of humidity on the σdc of the soPA membrane was smaller than for the sbPA membrane, and even at 80 °C and 10% RH, the σdc of soPA reached 0.66 mS cm^–1^, which was approximately ten times higher than that of sbPA..

**Figure 6:**
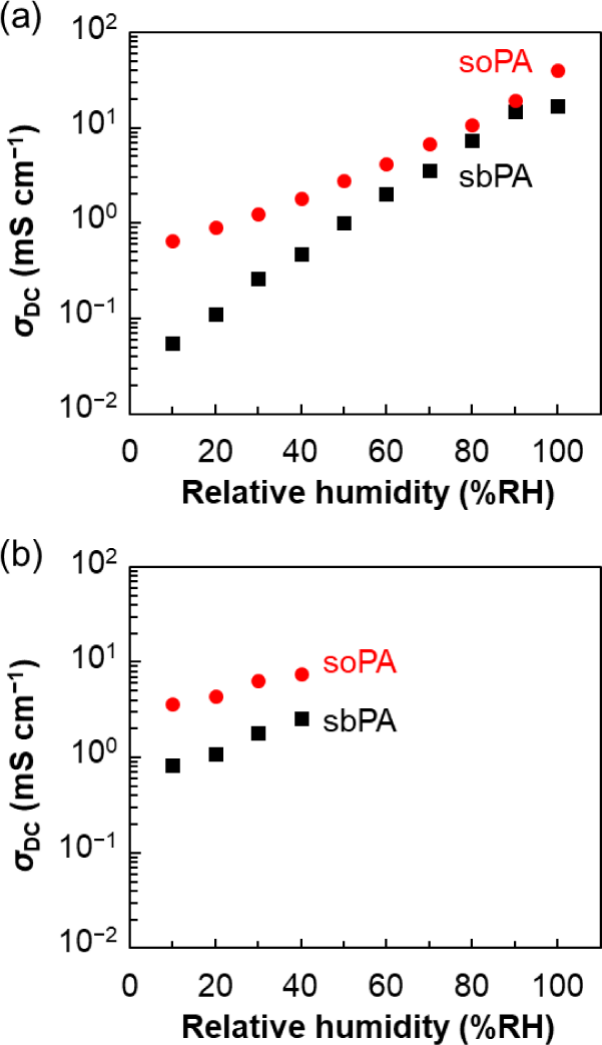
Relative humidity dependence of the conductivity of soPA (red circles) and sbPA (black squares) membranes at (a) 80 °C, and (b) 120 °C. See also ref 51 for conductivity of the sbPA membrane.

Although these data alone have limitations in fully elucidating the origin of the higher conductivities of soPA than those of sbPA, we discuss several possible factors based on established findings. The present result, in which the polymer with longer alkylene spacers exhibited higher conductivities, contrasts with a previous report on oligosiloxanes bearing phosphonic acid groups and alkylene spacers, where materials with shorter spacers showed higher conductivities.^70^ On the other hand, those oligosiloxanes did not form phase-separated structures, while the polymers investigated in this work formed aggregation of phosphonic acid groups and the clear nanophase-separated structure. Furthermore, the soPA membrane possessed higher morphological and molecular ordering of the nanophase separation than the sbPA membrane, and the relatively well-oriented lamellar structure of the soPA membrane is also likely to facilitate the uniform formation of hydrophilic channels.^71–73^ Such ordering-derived effects probably contributed to the enhanced conductivities of soPA. In addition, within the hydrophilic channels generated by the nanophase separation, the phosphonic acid groups may form “frustrated” hydrogen-bond networks, in which the numbers of proton donors and acceptors are imbalanced, thereby promoting proton conductivity.^74,75^

The good conductivities of soPA may also arise from the arrangement and dynamics of the phosphonic acid groups. Figure 7 shows conceptual illustration of the dynamics involved in the proton conduction mechanisms in soPA and sbPA. At temperatures below 100 °C and at high humidities, where sufficient water molecules were present, proton conduction in both soPA and sbPA proceeded mainly through proton hopping between water molecules and vehicle-type migration of water molecules that accept protons from the phosphonic acid groups. In addition, because the phosphonic acid groups were tethered via alkylene spacers and the side alkylene chains were flexible due to the absence of crystallinity, the acid groups were probably able to move in a vehicle-type manner, thereby contributing to proton transport. The longer alkylene spacers in soPA compared with sbPA allowed the phosphonic acid groups to move over a larger range, providing greater freedom of motion. Consequently, the conductivities of soPA were higher than those of sbPA under low temperatures and high humidities. Under low-humidity conditions, proton conduction mediated by water molecules is significantly suppressed due to the limited number of water molecules. Thus, proton transport predominantly relied on proton hopping between phosphonic acid groups and proton transfer associated with the motion of the acid groups themselves. The high degree of freedom in movement for the acid groups in soPA likely facilitated the proton transfer process (Figure 7), and the higher morphological and molecular ordering of nanophase separation occurred in the soPA membrane, resulting in less dependence on humidity and higher conductivities of soPA compared with sbPA, even under low-humidity conditions.

**Figure 7:**
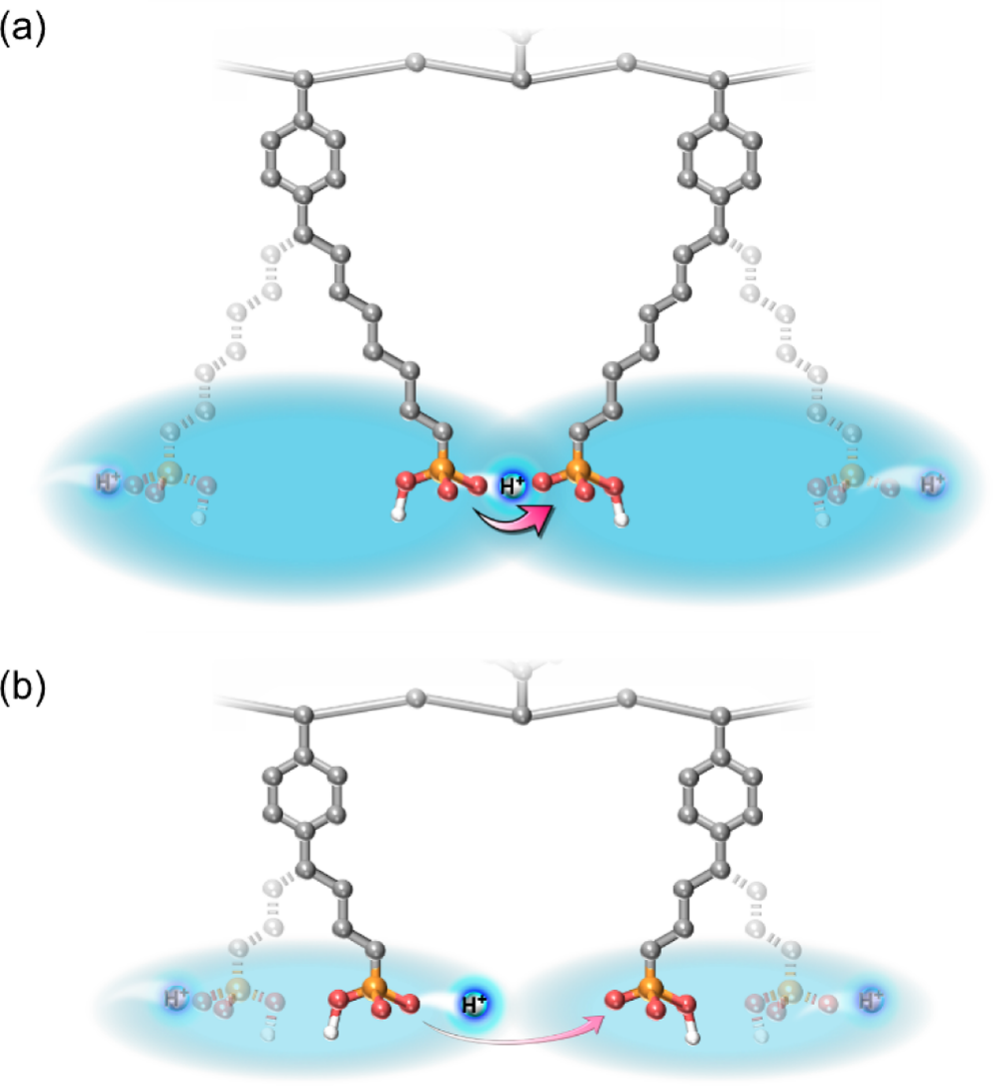
Conceptual illustration of the dynamics involved in proton conduction mechanisms in (a) soPA and (b) sbPA membranes at low relative humidity conditions.

The measurements of σdc at 120 °C, which is above the boiling point of water, were limited to 10–40% RH due to the difficulty of maintaining the pressurized environment required for high humidity. Under these conditions, the sbPA membrane exhibited σdc values of 0.83–2.6 mS cm^–1^,^51^ while the soPA membrane exhibited σdc values of 3.6–7.5 mS cm^–1^, which were 2.9–4.3 times higher than those of sbPA. As a preliminary experiment, we also synthesized the polymer with *n* = 6, namely poly­(6-(*p*-styryl)-1-hexanephosphonic acid) (shPA). It should be noted that the σdc values of shPA, with a six-carbon spacer, were 2.2–4.6 mS cm^–1^ at 120 °C and 10–40% RH, which was intermediate values between those of sbPA and soPA (see also Figure S8). This result supports the tendency that longer alkylene spacers lead to higher conductivity. Similar to the results at 80 °C, the superior σdcs of soPA under high-temperature and low-humidity conditions was attributed to the effect of the longer alkylene spacer groups. While the limited amount of available data does not permit a detailed discussion of humidity dependence at 120 °C, the humidity dependence of the σdc appeared to be weaker than that at 80 °C. This weak dependence was likely to be attributed to the increased molecular mobility of the alkylene spacers resulting from the 40 °C rise in temperature. To further inform the discussion on proton transport, we additionally examined the temperature dependence of conductivity at 30% RH, and estimated the apparent activation energy for proton transport in soPA and sbPA (Figure S9 and Table S2). While this analysis does not provide a complete mechanistic description, the lower activation energy observed for soPA compared with sbPA is consistent with the enhanced proton conductivity of soPA, and supports the view that longer alkylene spacers contribute to favorable transport characteristics. A more detailed mechanistic analysis will be pursued in future studies.

## 4 Conclusions

We synthesized soPA bearing phosphonic acid groups tethered through alkylene spacers with eight carbon atoms and evaluated the effect of spacer length on phase-separated structures and properties by comparing soPA with sbPA containing alkylene spacer groups with four carbon atoms to elucidate the structure–property relationship between spacer-length–dependent morphology and the resulting proton conductivity. When soaked in water at 60 °C for 3 h, the soPA membrane did not dissolve and exhibited a WU of 32% and a SR_*t*_ of 35%. TEM observation and X-ray scattering measurements revealed that soPA formed a well-oriented lamellar phase-separated structure with *D* = 2.9 nm, consisting of a hydrophobic phase containing the alkylene spacers and the polystyrene backbone, and a hydrophilic phase containing the phosphonic acid groups. Despite its lower acid group density, the soPA membrane exhibited higher proton conductivities at both 80 and 120 °C compared with sbPA. For example, at 80 °C, the soPA membrane exhibited conductivities of 0.66 mS cm^–1^ at 10% RH and 40 mS cm^–1^ at 100% RH, which are approximately 12 and 2.4 times higher than those of sbPA, respectively. Similarly, the soPA membrane exhibited moderate conductivities of 4.4 and 7.5 mS cm^–1^ at 120 °C under 20 and 40% RH, which are approximately 4 and 2.9 times higher than those of sbPA, respectively. These results can be attributed to the longer alkylene spacers in soPA, facilitating proton transfer between the phosphonic acid groups probably not only due to the higher degree of freedom in movement of the acid groups but also due to the higher morphological and molecular ordering compared with those in sbPA. In future studies, we will address the mechanical properties of the PEMs. We will also pursue a detailed deconvolution of the individual proton-conduction pathways (e.g., structural diffusion, vehicle transport) by quantifying the differences in proton mobility using variable-temperature ^1^H NMR measurements, measuring the conductivity in the nominally dry state,^34,70,76^ and evaluating the activation energy for proton transport at different relative humidities, and so on. In particular, conductivity measurements under nominally dry conditions in closed-cells will be an important focus in future study for investigation of the proton conduction mechanism. Although the orientation of the lamellar layers in the soPA membrane, which influences conductivity,^77,78^ has not been clearly determined in this study, it would be preferable for the lamellar layers to be oriented perpendicular to the membrane plane because PEMs are sandwiched between electrode layers when used in fuel cells; therefore, achieving or promoting a perpendicular orientation of the lamellar layers would be a desirable direction for future structural optimization. Overall, this study established the relationship between alkylene-spacer length, lamellar nanophase separation, and macroscopic proton conductivity, and demonstrates that the incorporation of longer spacer groups plays a crucial role in the molecular design of PEMs for next-generation PEFCs that require operation under high-temperature and low-humidity conditions.

## Supplementary Material



## Notes

^31^P NMR spectrum of monomer; GPC chromatogram of soPAdE; DSC thermogram of soPA; TGA thermograms of soPA; photos of the soPA membrane before and after water immersion; schematic illustration of the phase diagram for the phosphonic acid–based polymers; σdc values of the PEMs; activation energy for proton transport at 30% RH (Figures S1–S9 and Tables S1–S2) (PDF)
