# Supplementary material for: Long Alkylene Spacers Promote Structural Ordering and Proton Transport in Phosphonic Acid–Based Polymer Electrolyte Membranes
Source: ACS Omega. 2026 Jan 16;11(5):8272–82. doi: 10.1021/acsomega.5c10883 (PMC12902858; doi:10.1021/acsomega.5c10883)
Supplement: Supplementary file 1 [file ao5c10883_si_001.pdf]

Supporting Information for

Long Alkylene Spacers Promote Structural Ordering and Proton Transport in Phosphonic Acid-Based Polymer Electrolyte Membranes

Itsuki Takashima,[†] *Takato Kajita,*[†] *Takenori Nakayama,*[†] *Mio Nishimoto,*[†] *Haruka Tanaka,*[†] *Atsushi Noro*[*,†,‡,§]

[†]Department of Molecular & Macromolecular Chemistry, Graduate School of Engineering, Nagoya University, Furo-cho, Chikusa-ku, Nagoya 464-8603, Japan

[‡]Institute of Materials Innovation, Institutes of Innovation for Future Society, Nagoya University, Furo-cho, Chikusa-ku, Nagoya 464-8601, Japan

[§]Research Center for Net-Zero Carbon Society, Institutes of Innovation for Future Society, Nagoya University, Furo-cho, Chikusa-ku, Nagoya 464-8601, Japan

*E-mail: noro@nagoya-u.jp

^{31}P NMR spectrum of monomer.

Figure S1 shows the ^{31}P NMR spectrum of the product obtained in the second step of Scheme 1a. A peak at 33 ppm is attributed to the phosphorus atom of the phosphonate ester.

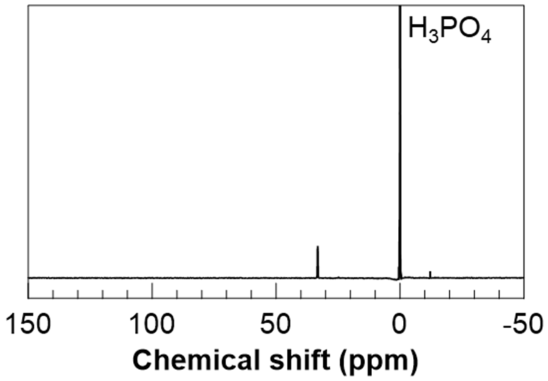

Figure S1. ^{31}P NMR spectrum of diethyl 8-(*p*-styryl)-1-octanephosphonate in $CDCl_3$.

GPC chromatogram of soPAdE.

Figure S2 shows the GPC chromatogram of soPAdE. The GPC chromatogram exhibits a unimodal distribution.

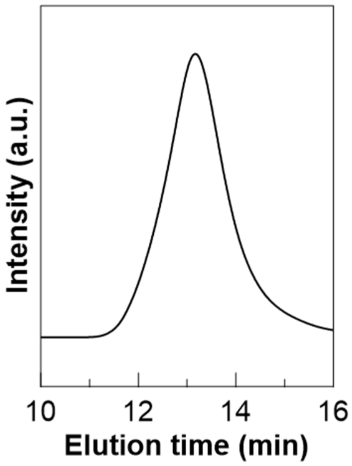

Figure S2. GPC chromatogram of soPAdE.

DSC thermogram of soPA.

Figure S3 shows DSC thermograms of soPA. T_g of soPA was determined to be 183 °C.

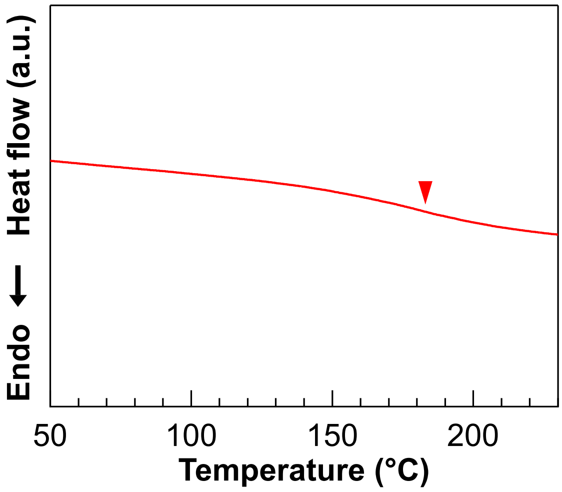

Figure S3. DSC thermograms of soPA, where the arrow indicates the positions of T_g.

TGA thermograms of soPA.

Thermal degradation of soPA was investigated by thermogravimetric analysis (TGA) using a DTG-60AH calorimeter (Shimadzu). The measurements were performed at a heating rate of 10 °C min^{-1} in the temperature range of 50–600 °C under a nitrogen atmosphere. The samples were sufficiently dried prior to the measurements. Figures S4a and S4b show TGA and differential thermogravimetry (DTG) curves, respectively. The weight loss of soPA was observed in three temperature ranges: approximately 50–180 °C, 180–360 °C, and 400–520 °C. The small initial weight loss between 50 and 180 °C, which is around the boiling point of water (≈100 °C), was attributed to evaporation of water absorbed in the polymer. The weight loss observed between 180 and 360 °C was primarily ascribed to dehydration condensation of the phosphonic acid groups. The third weight-loss event at the highest temperature range (400–520 °C) was probably attributed to degradation of the phosphonic acid groups and the polystyrene backbone. This thermal degradation behavior was similar to that reported for sbPA.[S1]

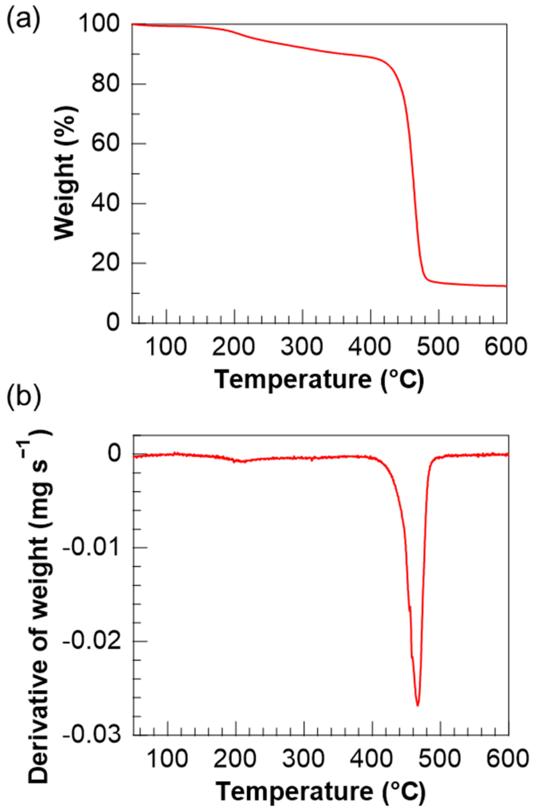

Figure S4. (a) TGA and (b) DTG thermograms of soPA.

Photos of the soPA membrane before and after water immersion.

Figure S5 shows images of the soPA membrane before and after the water immersion at 60 °C for 3 h. The soPA membrane did not dissolve in water.

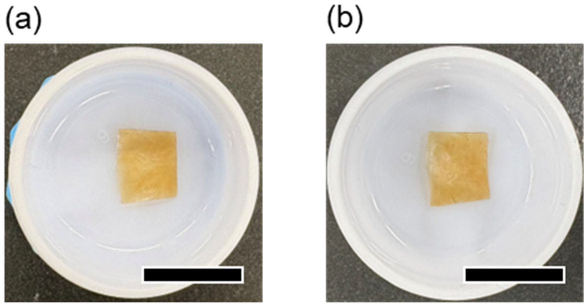

Figure S5. Photographs of the soPA membrane (a) before and (b) after immersion in water at 60 °C for 3 h; scale bars = 1 cm.

Schematic illustration of the phase diagram for the phosphonic acid-based polymers.

Figure S6 shows a schematic illustration of phase diagram for the phosphonic acid-based polymers. The diagram was prepared with reference to Figure 4 in ref. S2.

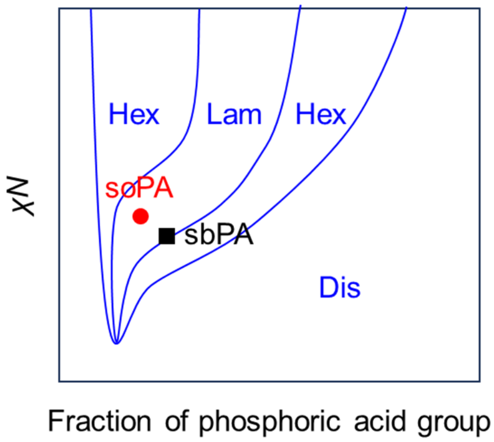

Figure S6. Schematic illustration of phase diagram for the phosphonic acid-based polymers. Hex, Lam, and Dis denote the hexagonal (cylindrical), lamellar, and disordered morphologies, respectively.

σ_{DC} values of the PEMs.

Table S1 summarizes the σ_{DC}s of the soPA and sbPA membranes at 80 °C and 120 °C. In measurements at 80 °C, relative humidity was reduced in 10% increments from 100 to 10%. For the measurements at 120 °C, the condition was first set to 100 °C and 100 %RH, then the temperature was raised to 120 °C, and the σ_{DC} was measured while decreasing the relative humidity stepwise from 40 to 10 %RH. Figure S7 shows the Nyquist plots for the PEMs. Bulk resistance (R), which corresponds to the resistance value under direct current conditions,[S3,S4] was estimated by reading the extrapolated intercept value on the Z' axis (the real part of the impedance) from the Nyquist plots, and the σ_{DC} was calculated using Equation S1, where A and l represent the cross-sectional area of the membrane and the distance between platinum electrodes, respectively.

$$\sigma_{\mathrm{DC}} = \frac{l}{AR} \tag{S1}$$

Figure S8 shows the σ_{DC}s of the poly(6-(*p*-styryl)-1-hexanephosphonic acid) (shPA) membrane at 120 °C. Table S1 also summarizes the σ_{DC} values of shPA. See also the discussion about the σ_{DC}s in Results and Discussion section.

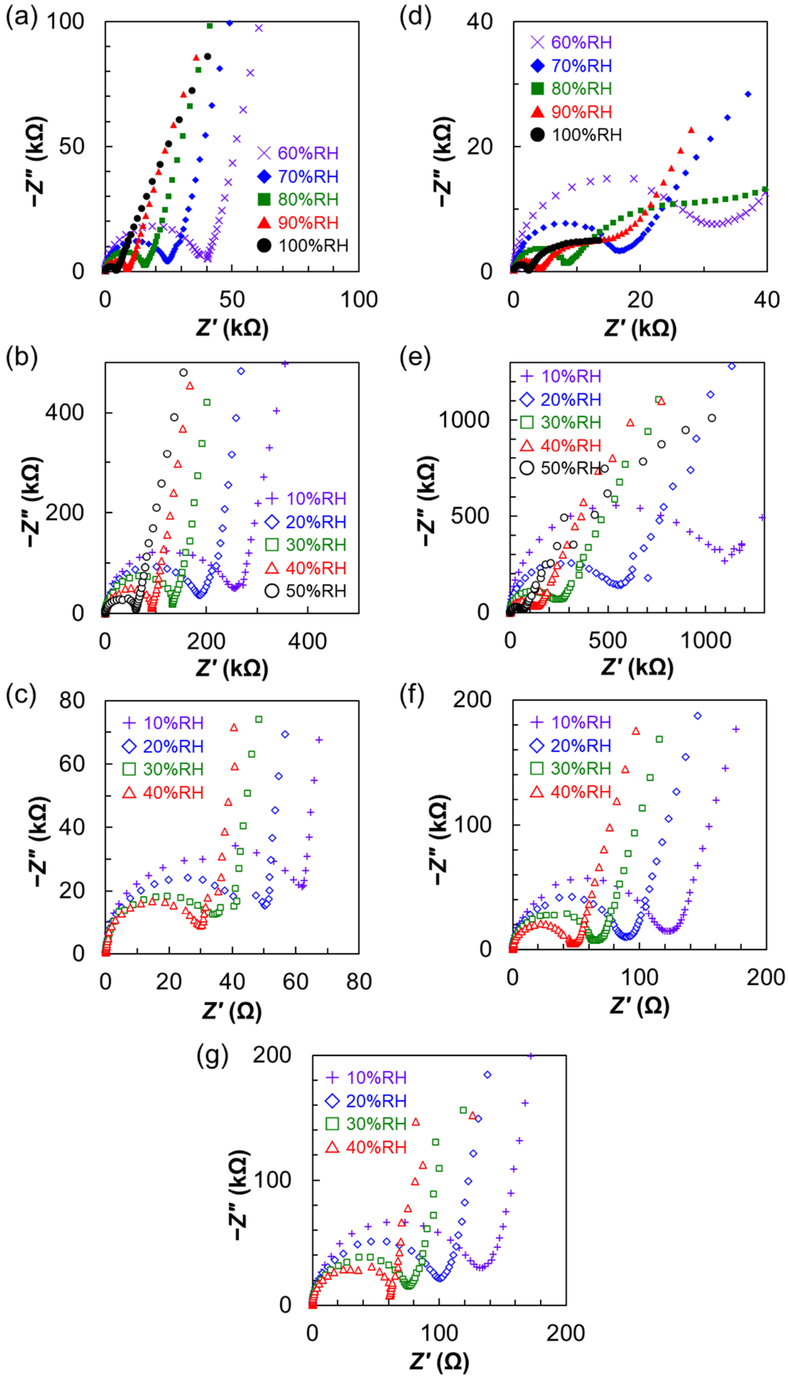

Figure S7. Nyquist plots for PEMs. The plots for soPA (a) at 80 °C under 60–100% RH, (b) at 80 °C under 10–50% RH, and (c) at 120 °C under 10–40% RH. The plots for sbPA (d) at 80 °C under 60–100% RH, (e) at 80 °C under 10–50% RH, and (f) at 120 °C under 10–40% RH. (g) The plots for shPA at 120 °C under 10–40% RH.

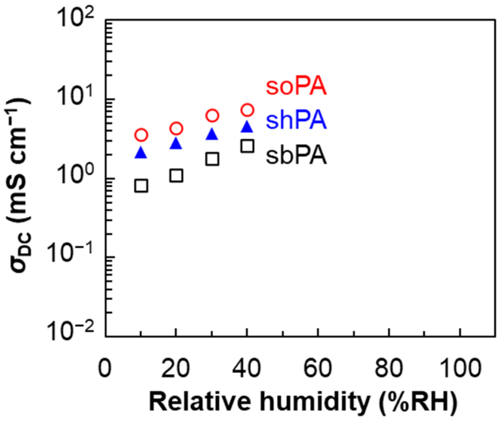

Figure S8. Relative humidity dependence of the conductivity of shPA membranes (filled blue triangles) at 120 °C. The conductivities of soPA (open red circle) and sbPA (open black square) membranes were also shown.

Table S1. Conductivities of PEMs

Temperature (°C)	Relative humidity (%RH)	σ_{DC} [a] (mS cm^{-1})		
		soPA	shPA	sbPA
80	100	40	–	17 [b]
	90	19	–	15 [b]
	80	11	–	7.4 [b]
	70	6.9	–	3.6 [b]
	60	4.2	–	2.0 [b]
	50	2.8	–	1.0 [b]
	40	1.8	–	0.47 [b]
	30	1.3	–	0.26 [b]
	20	0.90	–	0.11 [b]
	10	0.66	–	0.056 [b]
120	40	7.5	4.6	2.6 [b]
	30	6.4	3.7	1.8 [b]
	20	4.4	2.8	1.1 [b]
	10	3.6	2.2	0.83 [b]

[a]Conductivity determined by AC impedance spectroscopy.

[b]The data cited from ref. S1.

Activation energy for proton transport at 30% RH.

Figure S9 shows the Arrhenius plots of the conductivities and the Nyquist plots for soPA and sbPA membranes from 60 to 120 °C at 30% RH (soPA membrane: same polymer as discussed in the main text, prepared at a different time; sbPA membrane: prepared from a polymer with M_n = 75 kDa and $Đ$ = 3.1). For both soPA and sbPA, the logarithm of σ_{DC} was approximately proportional to the reciprocal of the absolute temperature (T) over the temperature range. In other words, the temperature dependence of the conductivity can be expressed by the Arrhenius equation (Equation S2) and the corresponding activation energies were evaluated on a provisional basis using the available data. In Equation S2, $\sigma_{DC,\infty}$, E_a, and R_g represent the conductivity when extrapolated to infinite temperature, the activation energy for proton transport, and the gas constant, respectively.

$$\sigma_{\mathrm{DC}} = \sigma_{\mathrm{DC},\infty} \exp\left(-\frac{E_\mathrm{a}}{R_\mathrm{g} T}\right) \tag{S2}$$

Table S2 summarizes the $\sigma_{DC,\infty}$, and E_a values of the soPA and sbPA membranes at 30% RH. Both the $\sigma_{DC,\infty}$, and E_a values of soPA were lower than those of sbPA. In this study, activation energies were evaluated only at 30% RH. A more comprehensive analysis over a wider humidity range will be addressed in future studies.

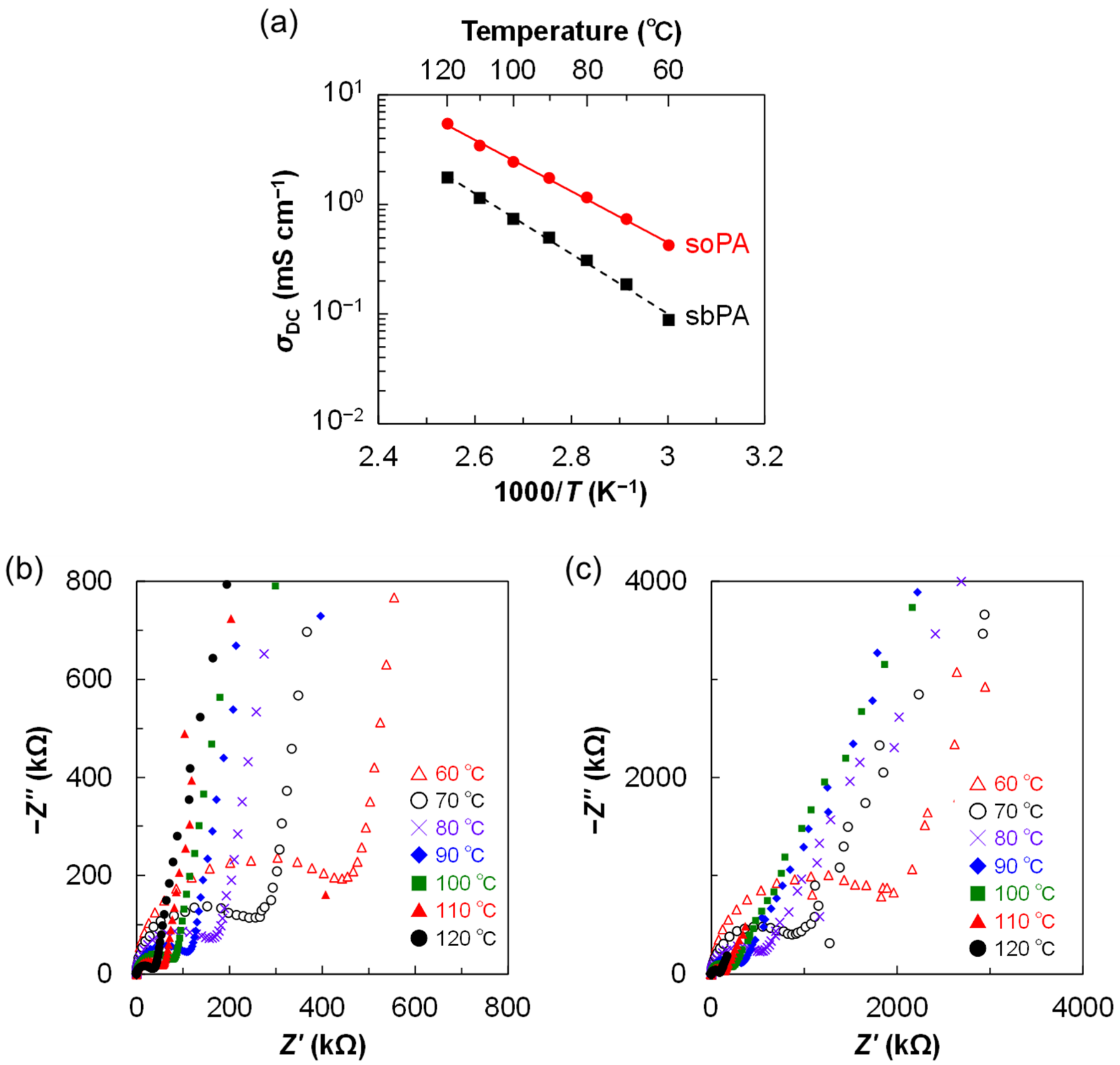

Figure S9. (a) Arrhenius plots of the conductivities for soPA (red circle) and sbPA (black square) membranes from 60 to 120 °C at 30% RH. The red solid (soPA) and black dashed (sbPA) lines represent fitting lines using the $\sigma_{DC,\infty}$ and E_a values. Nyquist plots for (b) soPA and (c) sbPA membranes from 60 to 120 °C at 30% RH.

Table S2. The $\sigma_{DC,\infty}$, and E_a values of the soPA and sbPA membranes at 30% RH

Sample	$\sigma_{DC,\infty}$ (mS cm^{-1})	E_a (kJ mol^{-1})
soPA	4.6×10^6	45
sbPA	1.9×10^7	53

References

(S1) Nakayama, T.; Kajita, T.; Nishimoto, M.; Tanaka, H.; Sato, K.; Marium, M.; Mufundirwa, A.; Iwamoto, H.; Noro, A. Polymer Electrolyte Membranes of Polystyrene with Directly Bonded Alkylenephosphonate Groups on the Side Chains. *ACS Appl. Polym. Mater.* **2024**, *6* (24), 15150–15161. https://doi.org/10.1021/acsapm.4c02688.

(S2) Sing, C. E.; Zwanikken, J. W.; Olvera de la Cruz, M. Electrostatic Control of Block Copolymer Morphology. *Nat. Mater.* **2014**, *13* (7), 694–698. https://doi.org/10.1038/nmat4001.

(S3) Bozkurt, A.; Ise, M.; Kreuer, K. D.; Meyer, W. H.; Wegner, G. Proton-Conducting Polymer Electrolytes Based on Phosphoric Acid. *Solid State Ion.* **1999**, *125* (1), 225–233. https://doi.org/10.1016/S0167-2738(99)00179-4.

(S4) Akgöl, Y.; Cramer, C.; Hofmann, C.; Karatas, Y.; Wiemhöfer, H.-D.; Schönhoff, M. Humidity-Dependent DC Conductivity of Polyelectrolyte Multilayers: Protons or Other Small Ions as Charge Carriers? *Macromolecules* **2010**, *43* (17), 7282–7287. https://doi.org/10.1021/ma1012489.
